# Magnetization reversal in trilayer structures consisting of GaMnAs layers with opposite signs of anisotropic magnetoresistance

**DOI:** 10.1038/s41598-018-20749-8

**Published:** 2018-02-02

**Authors:** Kyung Jae Lee, Sangyeop Lee, Seul-Ki Bac, Seonghoon Choi, Hakjoon Lee, Jihoon Chang, Suho Choi, Phunvira Chongthanaphisut, Sanghoon Lee, X. Liu, M. Dobrowolska, J. K. Furdyna

**Affiliations:** 1 Physics Department, Korea University, Seoul, 136-701 Korea; 2 Physics Department, University of Notre Dame, Notre Dame, IN 46556 USA

## Abstract

Magnetization reversal in a GaMnAs trilayer system consisting of two GaMnAs layers separated by a Be-doped GaAs spacer was investigated by magnetotransport measurements. The rotation of magnetization in the two GaMnAs layers is observed as two abrupt independent transitions in planar Hall resistance (PHR). Interestingly, one GaMnAs layer manifests a positive change in PHR, while the other layer shows a negative change for the same rotation of magnetization. Such opposite behavior of the two layers indicates that anisotropic magnetoresistance (AMR) has opposite signs in the two GaMnAs layers. Owing to this opposite behavior of AMR, we are able to identify the sequence of magnetic alignments in the two GaMnAs layers during magnetization reversal. The PHR signal can then be decomposed into two independent contributions, which reveal that the magnetic anisotropy of the GaMnAs layer with negative AMR is predominantly cubic, while it is predominantly uniaxial in the layer with positive AMR. This investigation suggests the ability of engineering the sign of AMR in GaMnAs multilayers, thus making it possible to obtain structures with multi-valued PHR, that can be used as multinary magnetic memory devices.

## Introduction

The alloy GaMnAs is a well known ferromagnetic semiconductor in which ferromagnetism is mediated by charge carriers^1–4^. Owing to the tunability of magnetic properties of this material by carrier density, it has received a great deal of attention in the field of spintronics. Dependences of Curie temperature, coercive field, and magnetic anisotropy on carrier concentration has already been thoroughly investigated in single-layer GaMnAs films^5–9^. Magnetic devices, however, typically involve combinations of two or more ferromagnetic layers, in which magnetic alignments switch their relative orientations, resulting in giant magnetoresistance (GMR) or tunneling magnetoresistance (TMR) effects^10–12^. In this context the process of magnetization reversal in GaMnAs-based multilayer systems needs to be thoroughly understood, in order to utilize such structures in spintronic device applications.

Several investigations addressing magnetization reversal in GaMnAs-based multilayers have already been carried out, reporting spin-valve behavior and/or interlayer exchange coupling in these systems^13–16^. All those investigations, however, were carried out on GaMnAs multilayers in which the anisotropic magnetoresistance (AMR) of individual GaMnAs layers have the same sign. In that case the planar Hall resistance (PHR) always changes in the same direction (positive or negative) in both GaMnAs layers as their magnetizations make transitions between adjacent quadrants, even when the coercive fields of the two layers are different. Understanding of the behavior of PHR during magnetization reversal in such GaMnAs multilayers is thus rather straightforward. However, the PHR behavior is expected to be significantly more complicated in GaMnAs multilayers in which individual GaMnAs layers have AMR with different signs, since changes in PHR depend on the sign of AMR as the magnetization rotates between adjacent quadrants. Even though most GaMnAs layers investigated so far show negative values of AMR^17^, observation of positive AMR has been reported in special cases when the flow of current in the GaMnAs film is oriented along carefully chosen crystallographic directions^18–20^, and in a Li-doped GaMnAs layer^21^. However, a GaMnAs trilayer structure with two opposite signs of AMR has never been fabricated, and transport properties during the magnetization reversal have never been investigated.

In the present study we observe both signs of AMR (i.e., negative and positive) in trilayers consisting of two GaMnAs layers separated by a heavily Be-doped GaAs spacer. Surprisingly, in such structures the AMR in the two GaMnAs layers exhibits opposite sign, revealed by opposite changes of the value of PHR as magnetization rotates in the same sense during both field and angular scans. A detailed investigation of magnetization reversal in this trilayer structure was performed by using a specific initialization of magnetization prior to measurement, described in the next section. By careful experiments and their analysis, we were then able to identify the magnetization alignments in the two magnetic layers, and to separate the contribution to PHR from each layer, thus allowing us to investigate the magnetic properties of the individual GaMnAs layers displaying the two signs of AMR.

## Experiment

**Figure 1:**
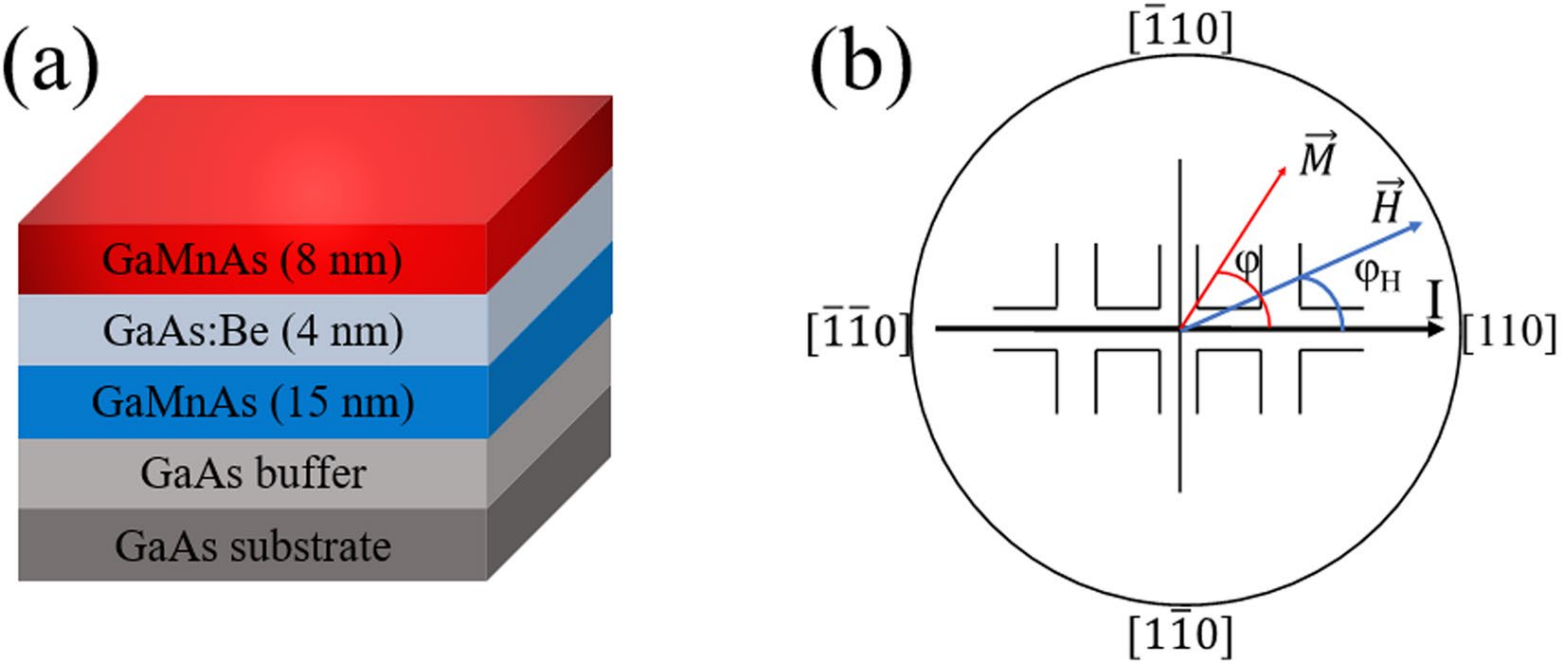
(**a**) Schematic diagram of trilayer structure grown on GaAs substrate. (**b**) Schematic diagram of the Hall device used for PHR measurements, with current flow along the [110] direction. Directions of current, external field, and magnetization are shown by arrows.

Two GaMnAs/GaAs:Be/GaMnAs trilayer structures used in this investigation were grown by molecular beam epitaxy (MBE) on (001) GaAs substrates. Prior to growth of the GaMnAs layer, a 100 nm GaAs buffer was deposited on the substrate at 600 °C. For deposition of GaMnAs layers, the growth temperature was lowered to 250 °C. A 15 nm bottom Ga_1*−x*_Mn_*x*_As film with *x* = 0.06 was first grown directly on the GaAs buffer, followed by deposition of a 4 nm GaAs spacer layer. During spacer deposition, Be was additionally supplied for p-type doping of the spacer. Two different temperatures, 1120° and 1140°, were used for the Be effusion cell in order to vary the doping level of the GaAs layer in the two trilayer structures, which we will refer to as samples T1 and T2, respectively. Finally, the top Ga_1*−x*_Mn_*x*_As film with the same of *x* as the bottom layer was deposited to a thickness of 8 nm. In addition, a single layer of GaMnAs, which we will refer to as sample S1, was grown with the same value of *x* as that used for trilayer growth, to be used as a reference sample. A schematic of the complete trilayer structure is shown in Fig. 1a.

For transport measurements, a 1000 × 10 μm^2^ Hall bar device was patterned on the specimen by photolithography and dry etching, with its long dimension along the [110] direction. A schematic view of the Hall device is shown in Fig. 1b. PHR measurements were performed using a sample holder designed so as to allow a magnetic field to be applied in arbitrary directions in the plane of the sample. The electromagnet used for this purpose was mounted on a rotating table, so that the field could either be swept along an arbitrary fixed direction, or it could be continuously rotated in the film plane with a fixed field magnitude. The directions of the applied magnetic field ${\phi }_{H}$ and of the magnetization ${\phi }_{M}$ of the magnetic layers are measured in the (001) crystal plane, counterclockwise from the [110] crystallographic direction (i.e., from the positive current direction of the Hall device), as shown in Fig. 1b.

## Results

### Magnetic field dependence of PHE

**Figure 2:**
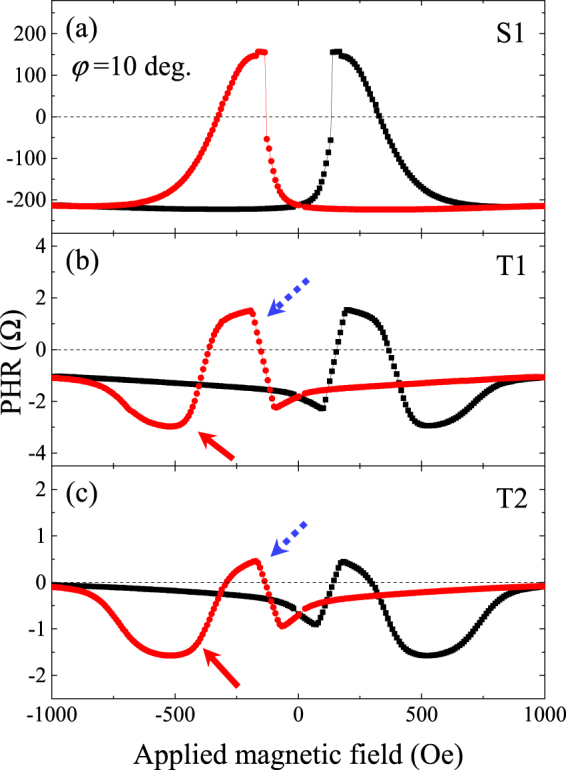
Planar Hall resistance obtained from three samples at 3 K at field orientation ${\phi }_{H}=10^\circ$. The data plotted in panels (**a**), (**b**), and (**c**) are obtained from samples S1, T1, and T2, respectively. Red and black symbols represent data obtained for down and up sweep of fields, respectively. Blue (dotted) and red (solid) arrows indicate downward and upward jumps showing hysteretic behavior, as discussed in the text.

Planar Hall effect (PHE) measurements are convenient for investigating magnetization reversal in ferromagnetic films, because they are very sensitive to the relative orientation of magnetization with respect to the current. This can be done by measuring transverse resistance *R*_*PHR*_ of the device shown in Fig. 1(b) in an external magnetic field, which can be either swept or rotated. We first performed field scan measurements of planar Hall resistance (PHR) at a fixed field direction in the film plane. Figure 2 shows the PHR results for the single GaMnAs layer (sample S1) and for the two trilayers (samples T1 and T2) obtained by scanning the field between +1000 Oe and −1000 Oe at ${\phi }_{H}=10^\circ$ orientation. The PHR data obtained on all samples show abrupt transitions as the field is swept, indicating that the magnetizations of the GaMnAs layers experience reorientations between easy axes in the film plane. For such transitions of magnetization, the behavior of PHR is described by the equation^7,22,23^

1 $${R}_{PHR}(M,J;{\phi }_{M})={\rm{\Delta }}R\,\sin \,2{\phi }_{M},$$

where *R*_*PHR*_ is the value of the planar Hall resistance, *M* is the magnetization, *J* is the current density, ${\phi }_{M}$ is the angle between *J* and *M*, and Δ*R* is the anisotropic magnetoresistance (AMR) parameter defined as ${\rm{\Delta }}R=({R}_{||}-{R}_{\perp })$, with ${R}_{||}$ and ${R}_{\perp }$ representing resistances when magnetization is parallel and perpendicular to the current flow, respectively.

It is well known that for GaMnAs films grown on GaAs substrates the parameter Δ*R* is typically negative^7,17^. In that case the value of *R*_*PHR*_ is negative when the magnetization is either in the 1^st^ or the 3^rd^ quadrants (i.e., when $0^\circ  < {\phi }_{M} < 90^\circ$ or $180^\circ  < {\phi }_{M} < 270^\circ$), while it is positive when the magnetization is in the 2^nd^ or the 4^th^ quadrants (i.e., when ${\rm{90}}^\circ  < {\phi }_{M} < 180^\circ$ or $270^\circ  < {\phi }_{M} < 360^\circ$). The PHR of sample S1 plotted in the top panel shows negative values at saturation (i.e., when the magnetization is oriented in the 1^st^ and in the 3^rd^ quadrants by the strong external field), but during magnetization reversal experiences transitions to positive values as the magnetization rotates to its interim easy axes in the 2^nd^ and the 4^th^ quadrants (see low field regions in Fig. 2a). This behavior of PHR indicates the presence of two in-plane magnetic easy axes near the $\langle 100\rangle$ directions, as well as a negative value of Δ*R*, typical for single GaMnAs layers.

The PHR results obtained for samples T1 and T2 are plotted in Fig. 2b,c, and show a very different behavior from sample S1. In a trilayer comprised of two GaMnAs layers with the same sign of Δ*R* one would expect twice as many transition steps as in S1 due to different coercive fields of the two magnetic layers. In that case both layers would contribute to PHR in the form of changes in the same direction (both up, or both down) for the same sense of magnetization rotation at the coercive fields of the two layers^14^. Samples T1 and T2 indeed do show additional transition steps during magnetization reversal, but the observed behavior of PHR is quite different from that described above for a trilayer consisting of two GaMnAs layers with the same sign of Δ*R*. Specifically, the data plotted in Fig. 2b,c clearly show hysteretic behavior in which a increase of PHR (marked by blue arrow) is followed by an decrease (red arrow) below saturate value during reversal process. This indicates that the contributions to PHR due to each layer have *opposite* signs as the magnetization rotates in the same sense in the two layers, a process that can only be understood if Δ*R* in the two GaMnAs layers has opposite signs.

**Figure 3:**
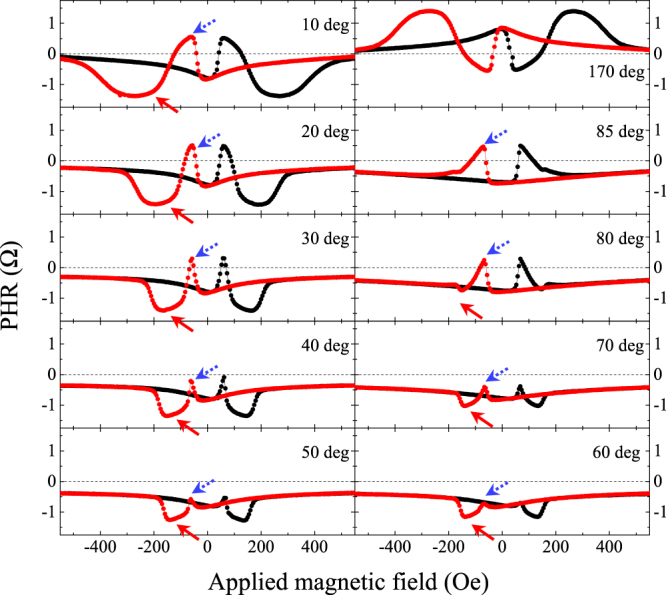
Planar Hall resistance obtained for sample T2 at 3 K. The field orientation was changed from ${\phi }_{H}=10^\circ$ to ${\phi }_{H}=85^\circ$ in successive panels. PHR data obtained at 170° is plotted at the top of the right column for comparison with the data obtained at 10° in the top panel of left column.

For a more detailed discussion of magnetization reversal in trilayers containing GaMnAs layers with opposite signs of ∆R, we will use the results obtained on sample T2. Figure 3 shows PHR data obtained on this sample by sweeping the field between +1000 Oe and −1000 Oe at ten different field directions. As already discussed in connection with Fig. 2b,c, the data in Fig. 3 are characterized by successive jump-like increases and decreases in PHR that occur as the magnetization reorients in the two layers with different signs of ∆R. Note the conspicuous changes in the upward and downward “jumps” in PHR (marked by dotted blue and solid red arrows, respectively) observed at different field orientations. The upward jumps, caused by the GaMnAs layer with negative ∆R, become systematically weaker as the field angle increases from 10° to 60°, at which they nearly disappear; and then begin to increase again as the field angle increases beyond 60°. Since the hysteresis in PHR is most narrow when the field is oriented along the easy axis^24,25^, from the behavior just described we conclude that the GaMnAs layer with negative ∆R has its magnetic easy axis near 60°, deviating about 15° from the [010] direction. This deviation provides a measure of the relative strengths of uniaxial anisotropy field Hu along $[1\bar{1}0]$ and cubic anisotropy field Hc along one of the $\langle 100\rangle$ directions to be Hu/Hc = 0.50 for the GaMnAs layer with negative ∆R^26^.

The negative jump of PHR, on the other hand, becomes continuously weaker as the field direction increases to 85°, at which angle it disappears (see the 2^nd^ panel in the right column of Fig. 3). This indicates that the GaMnAs layer with positive ∆R has its magnetic easy axis near the 85° orientation, close to the $[\bar{1}10]$ direction. This orientation of the easy axis provides the relative strength of uniaxial anisotropy field along the $[1\bar{1}0]$ direction to the cubic anisotropy field along the $\langle 100\rangle$ directions as Hu/Hc = 0.985 for this layer.

**Figure 4:**
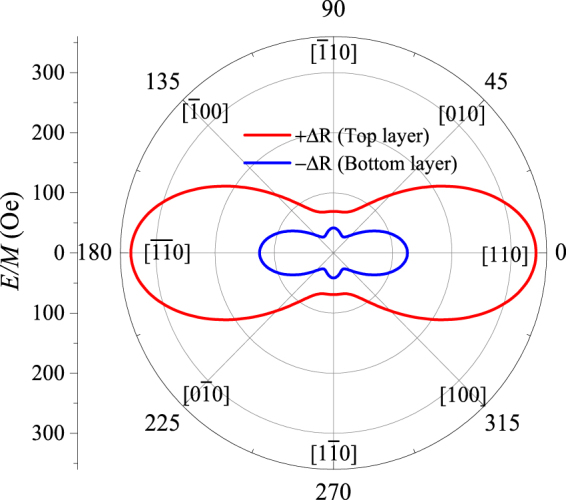
Magnetic free energy diagrams of the two GaMnAs layers with negative and positive ∆R, drawn with blue and red lines, respectively.

The PHR pattern observed at 170°, shown for completeness, is a perfect mirror image of the data obtained for 10° shown in the top left-hand panel of Fig. 3, indicating that the magnetic easy axes, and thus the magnetic anisotropy fields of the two GaMnAs layers, are symmetric with respect to the $\langle 110\rangle$ direction. The numerical values of magnetic anisotropy fields can be obtained by analyzing the angular dependence PHR, as discussed in Supplementary Information 1. Based on this analysis, we can draw the magnetic free energy diagrams for the two GaMnAs layers in the trilayer structure, as shown in Fig. 4, where the red and blue contours correspond to layers with positive and negative ∆R, respectively. The diagrams clearly show that the layer with positive ∆R is predominantly uniaxial, while the layer with negative ∆R has a strong admixture of cubic anisotropy, as evidence by four well-defined energy minima near the $\langle 100\rangle$ directions.

### Angular dependence of PHE

**Figure 5:**
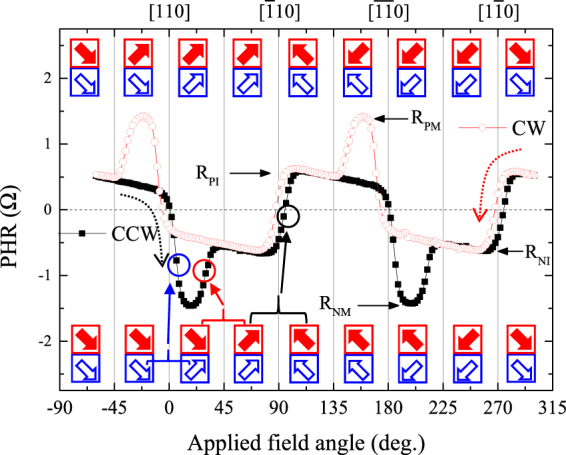
Angular dependences of PHR data obtained at 3 K by rotating magnetic a field of 200 Oe in the sample plane. The open and solid symbols show results obtained with clockwise (CW) and counterclockwise (CCW) field rotations, respectively. Thick solid (red) and open (blue) arrows indicate magnetization orientations in GaMnAs layers with positive and negative Δ*R*, respectively.

In order to follow how the magnetizations of GaMnAs layers with opposite sign of ∆R reorient during magnetization reversal, we performed angle-dependent measurements of PHR by rotating the field in the film plane at a fixed field magnitude. Representative angle-dependent PHR data obtained by rotating a field of 200 Oe over 360° is shown in Fig. 5, where the data for counterclockwise (CCW) rotation are plotted as black squares, and for clockwise (CW) rotation as red circles. In the figure we will schematically indicate the directions of magnetization at different field orientations by thick arrows, solid red arrows for magnetization in the GaMnAs layer with positive ∆R, and open blue arrows for the layer with negative ∆R. The succession of magnetization orientations shown in the top two rows of arrows is for CW rotation, and in the bottom two rows for the CCW rotation.

For PHR measurements shown in the figure, the magnetization of the trilayer (i.e., of both GaMnAs layers) was initialized by applying a field of 2000 Oe in the 4^th^ quadrant, as shown by the two thick arrows in the region between −90° to −45° in Fig. 5. The field was then reduced to 200 Oe, and the PHR was first measured as the direction of the field was rotated CCW from −60° to 300°. The observed PHR shows several abrupt transitions. The first two transitions, occurring between 0° to 45° (first downward, and then upward) are caused by consecutive rotations of magnetization from the 4^th^ quadrant to the 1^st^ in the two GaMnAs layers. The steep downward drop occurring right after 0° to negative values of PHR (black symbols) corresponds to rotation of magnetization in the GaMnAs layer with negative Δ*R*, as inferred from Eq. (1). The upward transition that follows, occurring before 45° and representing a positive contribution to PHR, is due to the rotation of magnetization in the GaMnAs layer with positive Δ*R*. The angular positions at which these successive rotations occur are marked by arrows below the plots. This sequence of magnetization transitions for the two GaMnAs layers with opposite signs of Δ*R* is consistent with the larger (smaller) coercive fields for the GaMnAs layer with the positive (negative) sign of Δ*R* observed in field scan measurements shown in Figs 2 and 3.

As the field direction passes 90° during CCW rotation, the PHR jumps abruptly to a positive value. This is a result of magnetization rotation from the 1^st^ to the 2^nd^ quadrant in both GaMnAs layers (see thick arrows at bottom of figure). The change in PHR for this simultaneous transition in the two GaMnAs layers is smaller than that caused by the transition in the single GaMnAs layer with negative Δ*R*, since it is a superposition of opposite contributions to PHR from GaMnAs layers with opposite signs of Δ*R* as the magnetization rotates from the 1^st^ to the 2^nd^ quadrant. Thus PHR reaches only an intermediate value (marked R_PI_), which is a stable state with parallel orientations of magnetization in the trilayer, as indicated by thick arrows at bottom of the figure. When the field direction is further rotated CCW, past 180°, the GaMnAs layer with the negative sign of Δ*R* rotates first from the 2^nd^ quadrant to the 3^rd^ (open arrows), resulting in perpendicular alignment of magnetizations in the two magnetic layers. In this configuration the PHR of the trilayer reaches a minimum value, marked by R_NM_. The magnetization of the GaMnAs layer with the positive Δ*R* undergoes the same rotation at a slightly higher angle, resulting in an intermediate PHR value marked R_NI_. Finally, as the field orientation passes 270°, the magnetizations of both layers again rotate together (from the 3^rd^ to the 4^th^ quadrant), returning to the initial configuration, as shown by the arrows (and thus returning to the PHR value of R_PI_).

Note further that, in the behavior just described, changes in PHR caused by the GaMnAs layer with negative Δ*R* (e.g., the first jump right after 0°) are about twice as large as those caused by the layer with positive Δ*R* (e.g., the jump just below 45°) for the same rotation of magnetization over the [110] direction. Since the bottom GaMnAs layer is about twice as thick as the top layer, it is expected to cause a larger contribution to PHR for the same rotation of magnetization. This allows us to identify the bottom GaMnAs layer of the trilayer structure as having negative Δ*R*, and the top layer as having Δ*R* with a positive sign.

When the field is rotated CW, the rotations of magnetization occur in reverse sequence, as shown by thick arrows in the top two rows of Fig. 5. The difference in the data obtained with CW rotation from the CCW data is that the PHR now exhibits positive maximum values, marked R_PM_ in Fig. 5, instead of negative, marked R_NM_. The value of R_PM_ and R_NM_ occur at field orientations when the magnetizations in the two GaMnAs layers are in perpendicular alignment. As indicated by the thick arrows in the figure, this alignment results in the pronounced PHR minima observed in the CCW rotation (black symbols), and in the pronounced maxima in the CW case. Importantly, such multiple PHR values realized by stable magnetic configurations of the two GaMnAs layers with opposite sign of Δ*R* provide an opportunity for applications as multi-valued storage devices^27,28^.

The concept for PHE-based memory applications can be also found in earlier literature^29,30^. We note, however, that the work of Bason *et al*.^29^ presents a concept of magnetic memory based on the PHE in a single magnetic layer with two in-plane magnetic easy axes; and the work by Sulaev *et al*.^30^ reports a continuous change of AMR in BiSbTeSe_2_ topological insulator by an applied gate electric field. In contrast, the multiple PHR states in our structure were realized based on various magnetic alignments in magnetic layers of the trilayer, clearly differing from the processes involves in those earlier investigations.

### Temperature dependence of PHR

**Figure 6:**
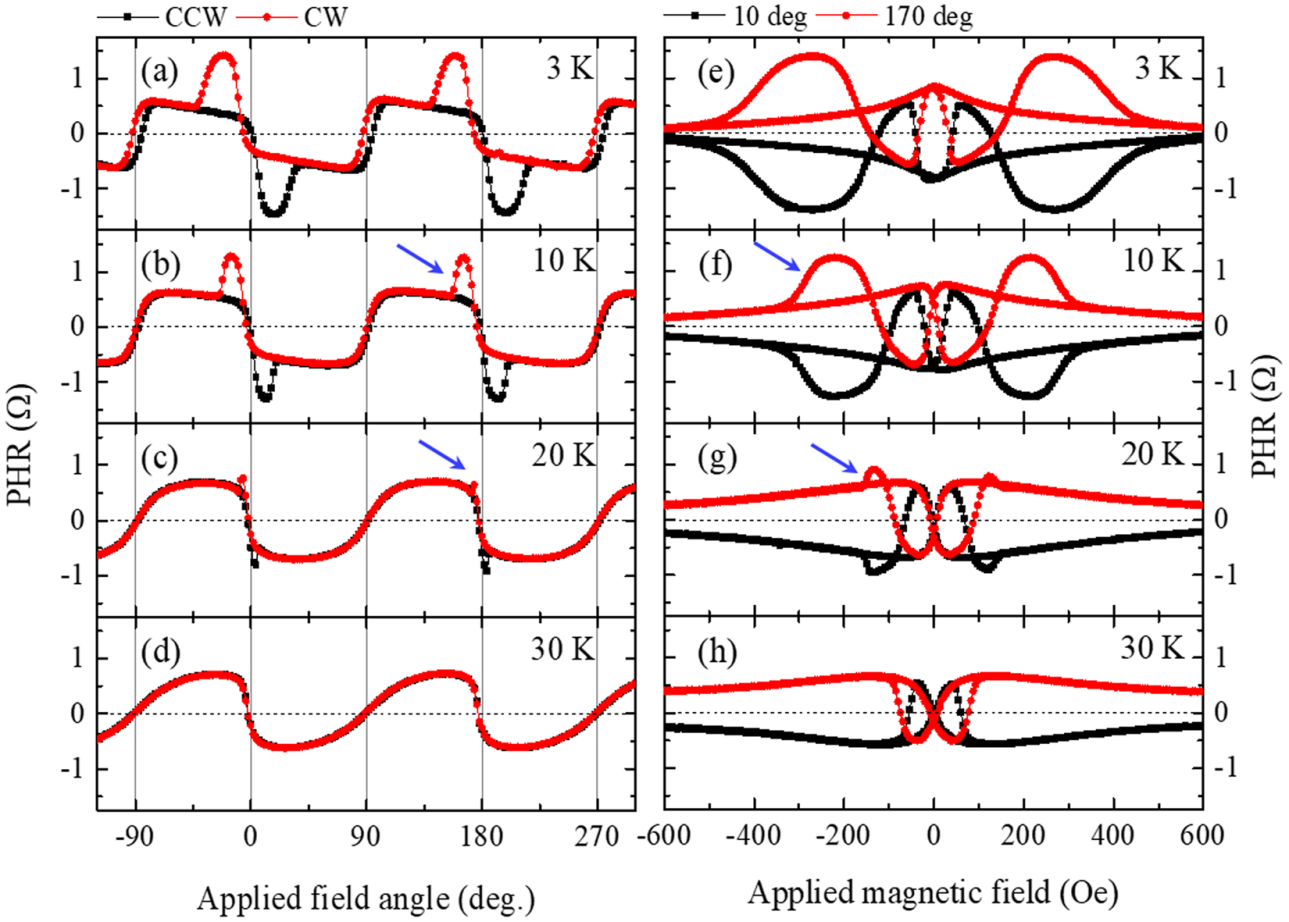
PHR data taken at four different temperatures. (**a**)–(**d**) Angular dependences of PHR taken by rotating a magnetic field of 200 Oe. Transitions appearing near 0° and 180° (i.e., near [110] and the $[\bar{1}\bar{1}0]$ directions) rapidly diminish with increasing temperature. (**e**)–(**f**) Field scans of Hall resistance obtained with magnetic field along 10° and 170°. The hysteresis showing a larger coercive field disappears with increasing temperature. In both measurements, the contribution from the GaMnAs layer with positive Δ*R* disappears at a lower temperature than that from the layer with negative Δ*R*. As is typical for GaMnAs, coercive fields associated with the observed hysteretic behavior diminish rapidly as the temperature increases.

To obtain additional understanding of the properties of GaMnAs layers in trilayer configuration, we also studied the dependence of PHR on temperature. PHR measurements of magnetization reversal obtained at several temperatures by sweeping the field are shown in the right-hand panels of Fig. 6, and PHR measured by rotating a field of constant magnitude are shown on the left of the figure. One can clearly see that the contribution to PHR from the layer with positive Δ*R* (see transitions marked by arrows) decreases very rapidly with increasing temperature in both types of measurement, and nearly disappears at 30 K, which is close to the Curie temperature of one of the GaMnAs layers in sample T2 (see resistance peaks observed in temperature scans for that sample shown in Fig. S1 in Supplementary Material 2). This observation indicates that the GaMnAs layer with a lower Tc and a larger coercive field has a positive Δ*R*, which we have already identified with the top layer of the trilayer structure. Here we should note that p-type doping significantly affects both the Curie temperature and the magnetic anisotropy of GaMnAs^31–33^. It is therefore likely that the p-type doping of the GaAs spacer is the cause of the reversed sign of Δ*R* in the top GaMnAs layer. The mechanism of such reversal of AMR is, however, not clear at this time, and further systematic investigation of this behavior is still necessary.

### Separation of contributions of top and bottom layer to PHR

**Figure 7:**
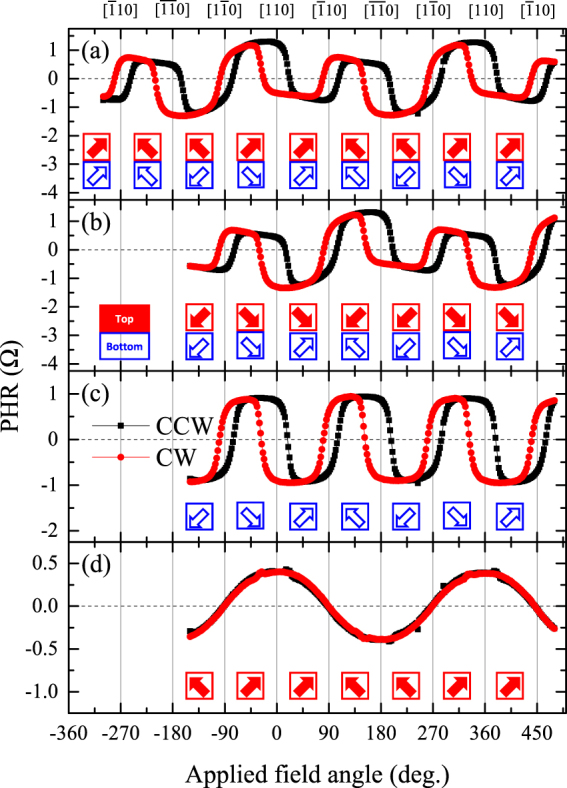
Angular dependences of PHR obtained after initializing magnetization (**a**) in the 1^st^ quadrant, and (**b**) in the 3^rd^ quadrant. (**c**) PHR signal of GaMnAs layer with negative Δ*R* (i.e., bottom layer of the structure) calculated from data in (**a**) and (**b**). The PHR signal from the GaMnAs layer with positive Δ*R* (i.e., the top layer) obtained by subtracting results in (**c**) from the PHR data in (**a**) is plotted in (**d**).

Since the observed PHR of the trilayer is a combination of contributions from the two constituent GaMnAs layers, we will consider these two layers as parallel conduction channels^34^ and will now attempt to separate and identify the contributions from each GaMnAs layer. We note from Fig. 4 that the two layers have very different magnetic anisotropies, which underlies the observed differences in their behavior, and can thus be exploited for separating the contributions of the two layers to PHR. For this purpose, we have performed a series of angle-dependent PHR measurements by using specific initialization procedures. Figure 7a,b show PHR data obtained with a field of 100 Oe after initialization of magnetization with an external field of 3,000 Oe applied along the directions in the 1^st^ and the 3^rd^ quadrants, respectively. Note that for this experiment, in contrast to angular studies described in the preceding section, we have chosen a rotating field of smaller magnitude, which (as will be seen) is sufficiently strong for overcoming the weak energy barriers in the GaMnAs layer with negative Δ*R* (see blue contour in Fig. 4), but is insufficient to overcome the much higher barriers characterizing the hard axes of the layer with a positive Δ*R* (red contour in Fig. 4). Following similar arguments to those used in describing Fig. 5, magnetic alignments of magnetizations in the two layers during rotation of the field are identified in Fig. 7 and shown as thick arrows at corresponding angular positions. As can be seen from the directions of the arrows, the GaMnAs layer with negative Δ*R* (already identified as the bottom layer) experiences a 360° rotation (see blue open arrows), while the layer with positive Δ*R* (i.e., the top layer) experiences only 90° rotations between adjacent quadrants (see red solid arrows). Such partial rotation of magnetization in the GaMnAs layer with positive Δ*R* is due to the large energy barriers along the [110] and $[\overline{11}0]$ directions shown in Fig. 4, which prevents continuous rotation of magnetization over 360° in that layer when a rotating field of only 100 Oe is used.

Owing to these specifics of magnetization rotation during reversal in the trilayer, we can now separate the contributions of the two GaMnAs layers to total PHR. For example, if we compare the PHR results in Fig. 7 obtained after initializing the magnetizations in the 1^st^ quadrant (Fig. 7a) with those obtained after magnetization was initialized in quadrant 3 (Fig. 7b), we note that the direction of magnetization of the GaMnAs layer with negative Δ*R* (blue open arrows in Fig. 7a,b) experiences exactly the same sequence of rotations over the entire 360° during reversal. Magnetization of the GaMnAs layer with positive Δ*R* (red solid arrows), on the other hand, experiences a different sequence of orientations when the measurement is initialized in quadrant 1 from that observed after initialization in quadrant 3. Since, as can be seen in Fig. 7, the magnetizations in that layer (solid red arrows) are symmetric with respect to the direction of the current, they result in contributions to PHR with the same magnitude but opposite sign (see Eq. (1)). The contribution of GaMnAs layer with positive Δ*R* to PHR can then be eliminated by adding the data in Fig. 7a,b, thus showing the net contribution of the GaMnAs layer with negative Δ*R*, which we plot in Fig. 7c. The data show an angular dependence of PHR typical for a GaMnAs layer with four in-plane hard magnetic axes along the 〈110〉 directions^35–38^. Note, however, that the hysteresis widths around the $[\bar{1}10]$ and $[1\bar{1}0]$ orientations are narrower than those around [110] and $[\overline{11}0]$, indicating the presence of uniaxial anisotropy along the $[\bar{1}10]$ and $[1\bar{1}0]$ directions. The observed angular dependence seen in Fig. 7c thus implies that the magnetic anisotropy of GaMnAs layer with negative Δ*R* is primarily cubic, but with a finite admixture of uniaxial anisotropy that makes the energy barriers along [110] and $[\overline{11}0]$ somewhat higher than those along $[\bar{1}10]$ and $[1\bar{1}0]$. This observation is consistent with the anisotropy of magnetic free energy shown in blue in Fig. 4.

Since the contribution of GaMnAs layer with negative Δ*R* to PHR is now established, it can be subtracted from the total trilayer PHR to obtain the contribution of the layer with positive Δ*R*. The angular dependence of PHR of the latter layer obtained by this procedure is plotted in Fig. 7d. Interestingly, the PHR of that layer shows no hysteresis between CW and CCW rotations, indicating that the rotation of magnetization in the layer with positive Δ*R* is limited to a coherent back-and-forth motion between the 1^st^ and 2^nd^ quadrants, as shown by red arrows in Fig. 7, since the field strength of 100 Oe selected for this experiment is insufficient to overcome the strong energy barriers between quadrants 2 and 3 (or between quadrants 1 and 4). This is because the GaMnAs layer with positive Δ*R* is almost entirely uniaxial (as already seen in Fig. 4). Note also in that figure that, while the magnetic energy barriers along [110] and $[\overline{11}0]$ are very high, those along $[1\bar{1}0]$ and $[\bar{1}10]$ are nearly negligible, thus allowing nearly coherent rotation of magnetization between the 1^st^ and 2^nd^ quadrants, as evidenced by the absence of a measurable hysteresis in Fig. 7d. The decomposition of angle-dependent PHR data into contributions from the two GaMnAs components of the trilayer thus fully confirms the magnetic anisotropy of two GaMnAs layers shown in Fig. 4.

## Summary

In summary, we have investigated the process of magnetization reversal of GaMnAs/GaAs:Be/GaMnAs trilayer structures using PHR measurements. We observed that in structures investigated the two constituent GaMnAs layers make contributions of opposite sign to the value of PHR for the same rotation of magnetization, thus indicating that the anisotropic magnetoresistance (AMR) has opposite signs in the two layers. Detailed investigation of the field and angular dependences of PHR obtained on the trilayer revealed that the sign reversal of AMR occurs in the top GaMnAs layer of the structure, which was deposited on a Be-doped GaAs spacer. The directions of magnetization in the two GaMnAs layers were carefully identified by analyzing the PHR data obtained during magnetization reversal. This identification of magnetic alignments has in turn allowed us to decompose the PHR signals of measured on the trilayer into separate contributions from the two GaMnAs layers. Interestingly, the GaMnAs layer with negative Δ*R* shows a typical cubic dominant magnetic anisotropy, with only a weak admixture of uniaxial anisotropy in the form of unequal energy barriers, while the layer with positive Δ*R* shows magnetic anisotropy that is almost entirely uniaxial, with high energy barriers along the [110] and $[\overline{11}0]$ crystallographic directions, and with nearly negligible barrier along $[1\bar{1}0]$ and $[\bar{1}10]$. This investigation shows the possibility of tuning the sign of AMR in GaMnAs layer, thus laying the ground for the realization of multiple PHR states via various magnetic alignments in GaMnAs-based trilayer structures. The demonstration of multiple stable states realized in the reversal process of our GaMnAs trilayer system with opposite signs of AMR is expected to stimulate similar investigations of systems based on other combinations of ferromagnetic materials, including systems operating at room temperature.

## Electronic supplementary material

Supplementary Information
