# Supplementary material for: Magnetization reversal in trilayer structures consisting of GaMnAs layers with opposite signs of anisotropic magnetoresistance
Source: Sci Rep. 2018 Feb 2;8:2288. doi: 10.1038/s41598-018-20749-8 (PMC5797254; doi:10.1038/s41598-018-20749-8)
Supplement: Supplementary file 1 — Supplementary Information [file 41598_2018_20749_MOESM1_ESM.pdf]

Magnetization reversal in trilayer structures consisting of GaMnAs layers with opposite signs of anisotropic magnetoresistance

- Supplementary Material -

Kyung Jae Lee[1], Sangyeop Lee[1], Seul-Ki Bac[1], Seonghoon Choi[1], Hakjoon Lee[1], Jihoon Chang[1], Suho Choi[1], Phunvira Chongthanaphisut[1], Sanghoon Lee[1*], X. Liu[2], M. Dobrowolska[2], and J. K. Furdyna[2]

[1]*Physics Department, Korea University, Seoul 136-701, Republic of Korea*

[2]*Physics Department, University of Notre Dame, Notre Dame, IN 46556, USA*

Supplementary 1

Magnetic anisotropy of the ferromagnetic semiconductor GaMnAs can be obtained by analyzing planar Hall resistance (PHR) data using the magnetic free energy equation given by[1]

$$F = M[\frac{H_C}{8}\cos^2 2\varphi_M + \frac{H_U}{2}\sin\varphi_M - H\cos(\varphi_M - \varphi_H)], \quad \text{(S1)}$$

where H_C is the in-plane four-fold symmetric cubic anisotropy field, H_U is the in-plane uniaxial anisotropy field, φ_M specifies the direction of magnetization in the GaMnAs film, and φ_H the direction of the applied magnetic field in the film plane. The angular positions of the energy minima in Eq. S1 at zero field, which can be found by applying conditions of $\frac{\partial F}{\partial \varphi_M} = 0$ and $\frac{\partial^2 F}{\partial {\varphi_M}^2} > 0$, specify the directions of magnetic easy axes of the specimen. The condition for the easy axes can then be written as[2] $\varphi_M = (\frac{1}{2})\sin^{-1}(\frac{H_U}{H_C})$. (S2)

Experimentally, the directions of easy axes in GaMnAs layers can be identified in field scan measurements of PHR, as described in the main text. In the present study the easy axes directions for the two GaMnAs layers in the trilayer turn out to lie near 60° and 85°, as measured from the [110] crystallographic direction. This gives the ratio of the uniaxial anisotropy field along the $[1\bar{1}0]$ direction Hu to the cubic anisotropy field along the <100> directions Hc as Hu/Hc = 0.500 and 0.985, respectively, for the GaMnAs layers with negative and positive ΔR.

Using these ratios of Hu/Hc, we can draw a qualitative shape of magnetic anisotropy for the two GaMnAs layers. To determine the magnetic anisotropy of the GaMnAs layers quantitatively, we performed angle-dependent measurements of PHR by using different external field strengths. Using the results of such experiments, we identify the field strength that is required to observe coherent rotation of magnetization,[3] which turns out to be 500 Oe for the GaMnAs layer with negative ΔR, and 1000 Oe for the layer in which ΔR is positive. By using these external fields for each GaMnAs layer, we calculated the energy profiles of the two layers using Eq. S1. We then systematically varied the magnitude of the anisotropy fields, to obtain a single common lowest minimum while the field direction is rotated. The progression of the energy diagrams with scanning field direction is shown in Fig. S1. Using this process, we obtained the values of magnetic anisotropy fields as Hu = 546 Oe and Hc = 554 Oe for the GaMnAs layer with positive ΔR, and as Hu = 167 Oe, Hc = 333 Oe for the layer with negative ΔR. By using these anisotropy values, a complete energy diagram can be drawn for each GaMnAs layer, as shown in Fig. 4 of the main text.

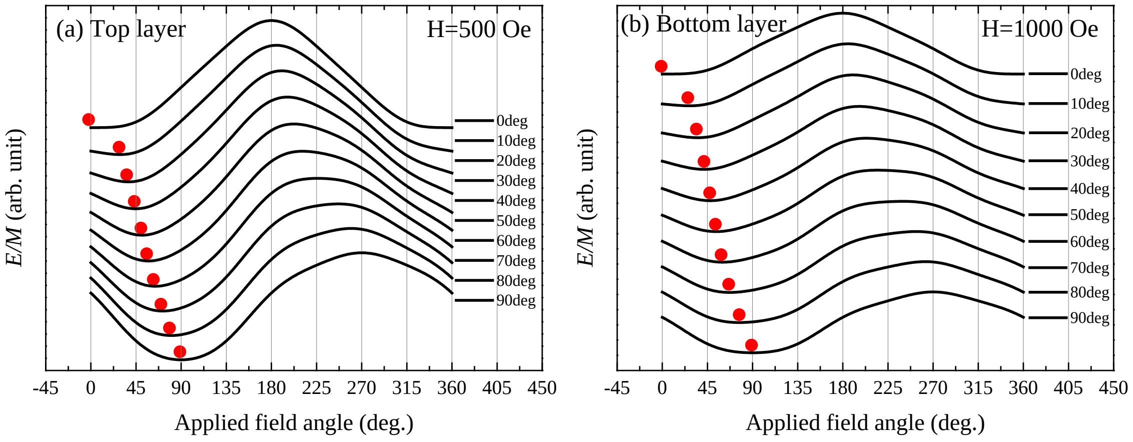

Fig. S1. Dependence of magnetic free energy on field direction for (a) the top and (b) the bottom GaMnAs layers. The minimum marked by a red point shows a continuous shift, indicating coherent rotation of magnetization with the field direction.

Supplementary 2

The two GaMnAs layers in the trilayer structure are expected to have different magnetic properties not only because of the difference in their thicknesses,[4] but also because the top GaMnAs layer is grown on a Be-doped GaAs, which is known to affect the magnetic properties of GaMnAs layer due to the presence of hole carriers.[5-7] In order to identify the Curie

temperatures of the two GaMnAs layers, we first measured the temperature dependence of the resistance of our trilayer specimens in the absence of a magnetic field. The resistance of sample T2 measured during the temperature scan down from 300 K to 3 K is plotted in Fig. S2. The data clearly show two resistance peaks, one around 35 K and one around 60 K, which provides an estimate of Curie temperatures of the two GaMnAs layers[8] comprising the trilayer structure. The observation of different Curie temperatures of the two layers suggests a difference in their magnetic anisotropies, and consequently a difference in magnetization reversal processes of the two layers.

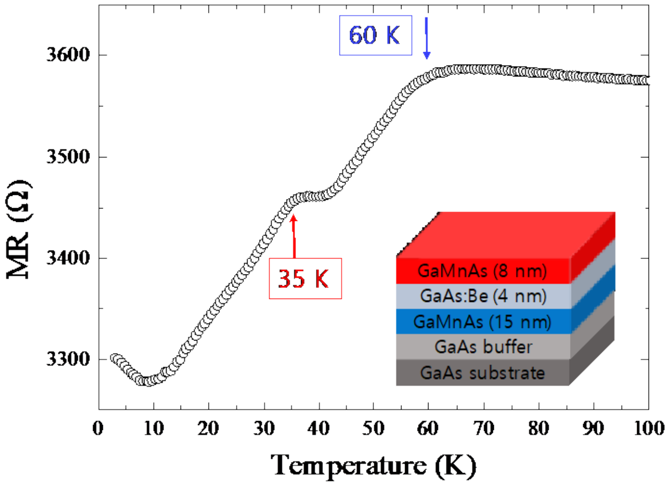

Fig. S2. Temperature dependence of resistance measured on sample T2. The two resistance maxima marked by arrows provide an estimate of the Curie temperatures of the two GaMnAs layers in the structure.

References

1 Shin, D. Y., Chung, S. J., Lee, S., Liu, X. & Furdyna, J. K. Temperature dependence of magnetic anisotropy in ferromagnetic (Ga,Mn)As films: Investigation by the planar Hall effect. *Physical Review B* **76**, doi:Artn 035327
10.1103/Physrevb.76.035327 (2007).

2 Tang, H. X., Kawakami, R. K., Awschalom, D. D. & Roukes, M. L. Giant planar Hall effect in epitaxial (Ga,Mn)As devices. *Physical review letters* **90**, 107201, doi:10.1103/PhysRevLett.90.107201 (2003).

3 E. C. Stoner, F. R. S. a. E. P. W. A Mechanism of Magnetic Hysteresis in Heterogeneous Alloys. *Philos. Trans. R. Soc. London, Ser. A* **240** (1948).

4 Bac, S. K. *et al.* Thickness dependence of uniaxial anisotropy fields in GaMnAs films. *Applied Physics Express* **8**, 033201, doi:Artn 033201
10.7567/Apex.8.033201 (2015).

5 Lee, S. *et al.* Effect of Be doping on the properties of GaMnAs ferromagnetic semiconductors. *Journal of Applied Physics* **93**, 8307-8309, doi:10.1063/1.1556272 (2003).

6 Wojtowicz, T. *et al.* Enhancement of Curie temperature in Ga1−xMnxAs/Ga1−yAlyAs ferromagnetic heterostructures by Be modulation doping. *Applied Physics Letters* **83**, 4220-4222, doi:10.1063/1.1628815 (2003).
7 Chung, S., Kim, H. C., Lee, S., Liu, X. & Furdyna, J. K. The effect of carrier density on magnetic anisotropy of the ferromagnetic semiconductor (Ga, Mn)As. *Solid State Communications* **149**, 1739-1742, doi:10.1016/j.ssc.2009.07.024 (2009).
8 Yuldashev, S. U. *et al.* Effect of additional nonmagnetic acceptor doping on the resistivity peak and the Curie temperature of Ga1−xMnxAs epitaxial layers. *Applied Physics Letters* **82**, 1206-1208, doi:10.1063/1.1554482 (2003).
